# RELIGIOSITY AND THE CONTROL OF DIABETES MELLITUS TREATMENT IN THE ELDERLY

**DOI:** 10.1093/geroni/igad104.2259

**Published:** 2023-12-21

**Authors:** Juarez Sales, Cris Renata Volpe, Luciano Lima, Marina Stival, Silvana Funghetto

**Affiliations:** University of Brasilia, Brasilia, Distrito Federal, Brazil; University of Brasilia, Brasilia, Distrito Federal, Brazil; University of Brasilia, Brasilia, Distrito Federal, Brazil; University of Brasilia, Brasilia, Distrito Federal, Brazil; University of Brasilia- Campus Ceilândia- UNB-Brasil, Brasília, Distrito Federal, Brazil

## Abstract

**Introduction:**

Diabetes Mellitus (DM) is an important health problem that affects thousands of Brazilians. Patients affected by the disease have many difficulties in following the correct treatment. Many studies point to religiosity as a factor that influences the health conditions of individuals. Objective: The study aimed to evaluate the influence of religiosity on diabetes mellitus treatment control in the elderly. Method: This is an outpatient-based explanatory cross-sectional study, in which a quantitative approach was taken. The study was conducted with 72 elderly people treated in primary health care in the Federal District. The instrument to evaluate the religiosity of the elderly was constructed through the defining characteristics and related factors of the NANDA-I nursing 6 diagnosis of impaired religiosity.

**Results:**

Environmental barrier to the practice of religion was found to be a risk factor for DM treatment uncontrolled.

**Conclusion:**

Religiosity plays an important role in the lives of the elderly, so it is becoming increasingly important to conduct research that assesses the importance of religion in the health-disease process of individuals. Nevertheless, nursing professionals must be attentive to this field of life attribute of the elderly and always encourage better relations of the patient with his religiosity.

